# Lack of CD8^+^ T-cell co-localization with Kaposi’s sarcoma-associated herpesvirus infected cells in Kaposi’s sarcoma tumors

**DOI:** 10.18632/oncotarget.27569

**Published:** 2020-04-28

**Authors:** Salum J. Lidenge, For Yue Tso, Owen Ngalamika, Jaydeep Kolape, John R. Ngowi, Julius Mwaiselage, Charles Wood, John T. West

**Affiliations:** ^1^Nebraska Center for Virology, Lincoln, NE, USA; ^2^School of Biological Sciences, University of Nebraska-Lincoln, Lincoln, NE, USA; ^3^Department of Biochemistry, University of Nebraska-Lincoln, Lincoln, NE, USA; ^4^Ocean Road Cancer Institute, Dar es Salaam, Tanzania; ^5^Muhimbili University of Health and Allied Sciences, Dar es Salaam, Tanzania; ^6^Dermatology and Venereology Section, University Teaching Hospitals, University of Zambia School of Medicine, Lusaka, Zambia; ^7^Morrison Microscopy Core Facility, Center for Biotechnology, University of Nebraska-Lincoln, Lincoln, NE, USA

**Keywords:** Kaposi’s sarcoma, HIV-1, KSHV, immune cells, tumor microenvironment

## Abstract

Despite the close association between Kaposi’s sarcoma (KS) and immune dysfunction, it remains unclear whether tumor infiltrating immune cells (TIIC), by their absence, presence, or dysfunction, are mechanistically correlated with KS pathogenesis. Therefore, their potential capacity to serve as prognostic biomarkers of KS disease progression or control is unclear. Because epidemic-KS (EpKS) occurs with HIV-1 co-infection, it is particularly important to compare TIIC between EpKS and HIV-negative African endemic-KS (EnKS) to dissect the roles of HIV-1 and Kaposi Sarcoma-associated herpesvirus (KSHV) in KS pathogenesis. This cross-sectional study of 13 advanced KS (4 EnKS, 9 EpKS) patients and 3 healthy controls utilized single-color immunohistochemistry and dual-color immunofluorescence assays to characterize and quantify KSHV infected cells in relation to various TIIC in KS biopsies. Analysis of variance (ANOVA) and Mann-Whitney tests were used to assess differences between groups where *P*-values < 0.05 were considered significant. The abundance of KSHV infected cells was heterogeneous in KS biopsies. Despite the presence of T-cell chemoattractant chemokine CxCL-9 in biopsies, CD8^+^ T-cells were sparsely distributed in regions with evident KSHV infected cells but were readily detectable in regions devoid of KSHV infected cells (*P* < 0.0001). CD68^+^ (M1) macrophages were evenly and diffusely distributed in KS biopsies, whereas, the majority of CD163^+^ (M2) macrophages were localized in regions devoid of KSHV infected cells (*P* < 0.0001). Overall, the poor immune cell infiltration or co-localization in KS biopsies independent of HIV-1 co-infection suggests a fundamental tumor immune evasion mechanism that warrants further investigation.

## INTRODUCTION

Increased incidence of Kaposi’s sarcoma (KS) in the HIV-1 infected (EpKS), transplant recipients, and in the elderly, implies that immune dysregulation or immune suppression plays a role in tumorigenesis [1, 2]. However, the precise nature of this dysregulation and how it drives KS remains poorly understood. Pathogenetic mechanisms driving African endemic-KS (EnKS), which is responsible for an estimated 4–10% of African adult cancers [3, 4], are even less clear, since EnKS occurs in both genders as well as children and in the absence of HIV-1 infection or other known immune dysfunctions. Indeed we have shown that KSHV viremia and humoral responses in EnKS show few significant differentials in comparison to those in EpKS [5].

Tumor infiltrating immune cells (TIIC) are indicators of tumor-related immune responses and are therefore often the targets of cancer immunotherapy [6]. For example, CD8^+^ T-cell infiltration in lung cancer and melanomas has been used as prognostic tool [7, 8]. In addition, tumor infiltration of myeloid lineage cells with a pro-inflammatory macrophage phenotype is also associated with tumor prognosis. Pro-inflammatory (M1) macrophages promote cytotoxic and anti-tumor CD8 and CD4 responses, whereas anti-inflammatory M2 macrophages contribute to Th2 responses, tissues repair and tumor growth [9–11]. Promoting a switch from the M2 to M1 phenotype in tumors and induction of pro-inflammatory cytotoxic T-cell responses has also been shown to slow or stop cancer growth [12]. Therapeutically, isolation of TIIC, such as T-cells, from tumors, followed by *ex vivo* expansion, antigenic stimulation and transfer back to the same patient is now a viable treatment strategy in cancers like melanoma and cervical carcinoma [13, 14]. Defining the value of TIIC as cancer prognostic marker is therefore an active area of research for a number of human cancers [7, 15, 16]. Nevertheless, despite the close association between KS and immune dysfunction [5], it remains unclear whether TIIC are a critical component in KS pathogenesis, and whether their absence, presence, or dysregulation could serve as a prognostic biomarker of KS disease progression or control. This is particularly relevant for comparison of EpKS to EnKS where the disease presentation, pathology and humoral immune parameters appear to be highly similar and therefore, the direct or indirect role of HIV-1 in KS remains unclear [5].

Our recent transcriptomic comparison of KS lesions to normal skin from the same individuals, revealed that KS lesions exhibited elevated expression of CxCL-9, CXCL-10 and CXCL-11 [17]. Since these chemokines are known to create chemotactic gradients for T-cell recruitment to sites of infection or loss of homeostasis [18], we asked whether CxCL-9 transcript upregulation was also evident at the protein levels in KS lesions, and if such over-expression correlated with immune cell infiltration into the KS microenvironment. Additionally, because transcriptomics revealed little or no HIV-1 transcription in EpKS lesions (16), we sought to investigate potential indirect effects of HIV-1 immune dysregulation in KS, through comparison of immune cell infiltration between EpKS and EnKS patients. We biopsied EpKS and EnKS patients from sub-Saharan Africa (SSA) to explore the relationships between chemokine expression, Kaposi’s sarcoma-associated herpesvirus (KSHV)-infected cells, TIIC and HIV-1 co-infection. Our study reveals poor immune cell infiltration in most KS tissues and lack of co-localization between TIIC and regions with demonstrable KSHV infection but detected no differentials in immune cell infiltration as a result of HIV-1 co-infection.

## RESULTS

### Characteristics of study subjects

To investigate the relationship between KSHV infected cells and TIIC in KS biopsies, samples with LANA+ cells demonstrable by IHC were utilized. A total of 13 KS cases (4 EnKS and 9 EpKS) and 3 normal skin donors were evaluated for this study. Ages in the cohort ranged from 27 to 84 with a median of 42 years (Table 1). The self-reported duration of KS ranged from 2 months to 3 years at the time of recruitment and was similar between EnKS and EpKS at a median of 6 and 3 months, respectively. EpKS patients were all ART experienced with undetectable plasma HIV-1 load, excepting patient C038 and 21242 who were on ART for less than a month and patient C3097 who was experiencing ART failure. Consistent with the most common presentation of KS in the region [19], most patients had nodular morphotype KS lesions on the extremities (Table 1).

**Table 1:** Characteristics of study subjects

ID	Gender	Age	HIV-1 Status	KS Duration^**^	ART Duration	Plasma HIV-1 load^*^	Lesion Type	Lesion Site
**C3096**	M	27	—	3 months	NA	NA	Nodular	L. Limb
**C3107**	M	84	—	2 years	NA	NA	Nodular	U. Limb
**C3138**	M	72	—	NR	NA	NA	Nodular	L. Limb
**C063**	M	58	—	6 months	NA	NA	Nodular	L. Limb
**C3057**	F	37	+	3 months	1 month	< 50	Plaque	L. Limb
**C3094**	M	45	+	2 months	2 years	< 50	Nodular	L. Limb
**C3097**	F	37	+	4 months	7 years	1.4E+06	Nodular	L. Limb
**C3104**	F	39	+	2 months	2 months	< 50	Nodular	L. Limb
**C216**	F	61	+	5 months	6 months	NR	Nodular	L. Limb
**C038**	F	44	+	3 months	2 days	1.5E+04	Nodular	Face
**21227**	M	40	+	2 months	3 months	< 50	Plaque	L. Limb
**21230**	M	34	+	3 years	3 years	< 50	Nodular	L. Limb
**21242**	M	44	+	3 months	21 days	3.2E+05	Nodular	L. Limb
**21650**	M	42	—			Normal Skin		
**21651**	M	42	—			Normal Skin		
**21652**	F	38	—			Normal Skin		

^*^copies/mL, ^**^Self-reported KS duration, L – Lower, U – Upper, NR – Not Recorded, NA – Not Applicable.

### Heterogeneous expression of LANA in KS biopsies

**Figure 1:**
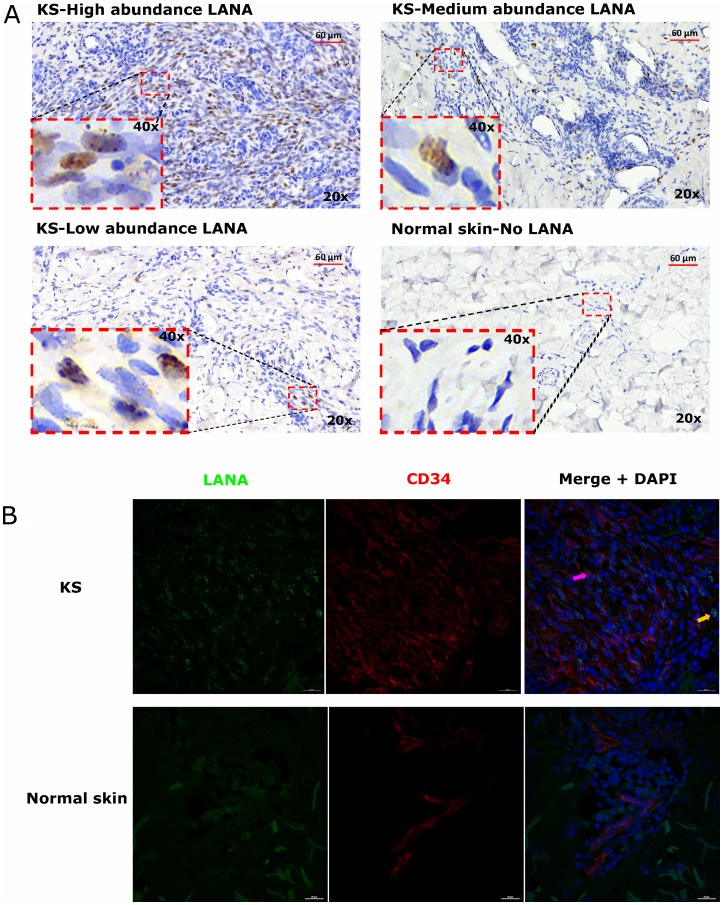
(**A**) Representative images showing varying abundancies of Kaposi’s Sarcoma-associated Herpesvirus (KSHV) Latency Associated Nuclear Antigen (LANA) protein on Kaposi’s Sarcoma (KS) tissues and normal skin by immunohistochemistry (IHC) staining. Representative images at 20× and 40× magnification are shown for KS tissue with high abundance LANA positive cells ID-21242, medium abundance LANA positive cells ID-21230, low abundance LANA positive cells ID-21229 and normal skin with no LANA positive cells ID-C063-control. (**B**) Dual-immunofluorescence staining of Kaposi’s Sarcoma-associated Herpesvirus (KSHV) Latency Associated Nuclear Antigen (LANA) protein and endothelial cell surface marker CD34 on Kaposi’s Sarcoma (KS) tissues ID-C3096 and normal skin ID-21650. Representative images are shown, Alexa 488 (green) for LANA, Alexa 647 (red) for CD34 and 4’,6-Diamino-2-Phenylindole, Dihydrochloride (DAPI) (blue) for nuclei staining. Pink arrow – LANA positive and CD34 positive cell, Orange arrow – LANA positive and CD34 negative cell.

To investigate the relationships between KSHV infected cells and TIIC, we first defined ‘infected cells’ as those evincing a punctate nuclear KSHV LANA staining pattern by IHC [20]. LANA is responsible for maintaining the KSHV episome in infected cells during latency and is also expressed in lytic reactivation [21]. In all KS biopsies, characteristic spindle-morphology cells were evident throughout the biopsy; however, we observed heterogeneous distributions of LANA^+^ cells. While LANA was not detectable in all spindle cells, some biopsies had a high abundance of LANA^+^ cells. KS biopsies with medium and low frequency LANA^+^ cell distributions were also common (Figure 1A).

### LANA+ cells in KS biopsies express endothelial cells marker

Studies have suggested that the majority of KSHV infected cells in tumors express markers of endothelial cell lineage including CD34 [22, 23]. To confirm the endothelial lineage of KSHV infected cells in the biopsies studied, we utilized DIF staining to simultaneously detect LANA in the nuclei and CD34 on the cell surface. We found that majority of the LANA^+^ cells in KS biopsies were CD34+ and thus appear to be of endothelial origin, whereas in normal skin, the CD34+ cells were confined to distinct vasculature and LANA was absent. Interestingly, although the majority of LANA^+^ cells appeared to be endothelial in origin, there were some CD34 negative but LANA^+^ cells, indicating that other cell types such as fibroblast, epithelial, dendritic and B cells demonstrated to support KSHV infection *in vitro* may also be present in KS tissues *in vivo* (Figure 1B) [24–26].

### KS tissues express chemoattractant CxCL-9

**Figure 2:**
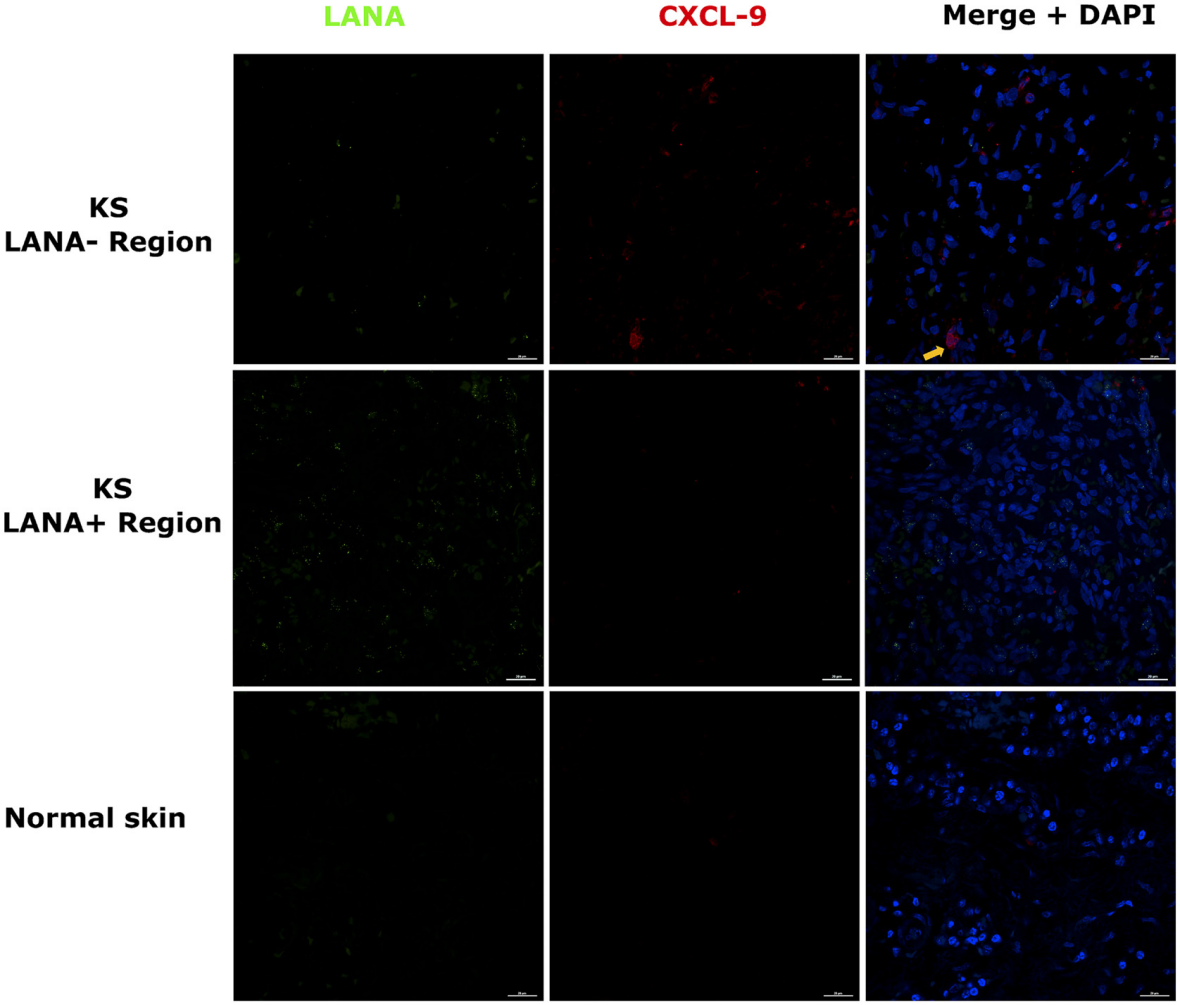
Dual-immunofluorescence staining of Kaposi’s sarcoma-associated Herpesvirus (KSHV) Latency Associated Nuclear Antigen (LANA) protein and C-X-C Motif Chemokine Ligand 9 (CxCL-9) on Kaposi’s Sarcoma (KS) tissues ID-C3057 (LANA+ and LANA- regions) and normal skin ID-21650. Representative images are shown, Alexa 488 (green) for LANA, Alexa 647 (red) for CxCL-9 and 4’,6-Diamino-2-Phenylindole, Dihydrochloride (DAPI) (blue) for nuclei staining. Orange arrow – CxCL-9 positive cell.

Chemokines create chemotactic gradients that can recruit immune cells to the sites of an infection or neoplasia [18]. Expression of T-cell chemoattractants in tissue could suggest an attempt to recruit T-cells to tissue sites. Alternately, these types of chemokines are often produced by myeloid cells that have polarized phenotypes that could be either cancer supportive, cancer repressive or neither. Our recent comparative transcriptomics analysis of KS biopsies versus ipsilateral/contralateral normal skin from the same individual demonstrated that KS lesions express significantly high levels of T-cell chemo-attractants CxCL-9, 10 and 11 compared to normal skin [17]. CxCL-9 was examined instead of CxCL-10 and -11 because it was upregulated the most in KS lesions vs controls skin at false discovery rate (FDR) < 5% [17]. Using DIF co-staining for LANA and CxCL-9, we found that CxCL-9 was indeed expressed at higher levels in both EpKS and EnKS tissue compared to normal skin, consistent with our published transcriptomics analysis [17] (Figure 2). Most CxCL-9 positive cells in KS tissue were LANA^-^ and localized in areas with reduced or undetectable LANA expression. Overall, this suggests that T-cell recruitment into KS is not localized to the KSHV infected regions of both EpKS and EnKS tissues.

### Lack of CD8^+^ T-cells co-localization with LANA-expressing cells in KS tissues

**Figure 3:**
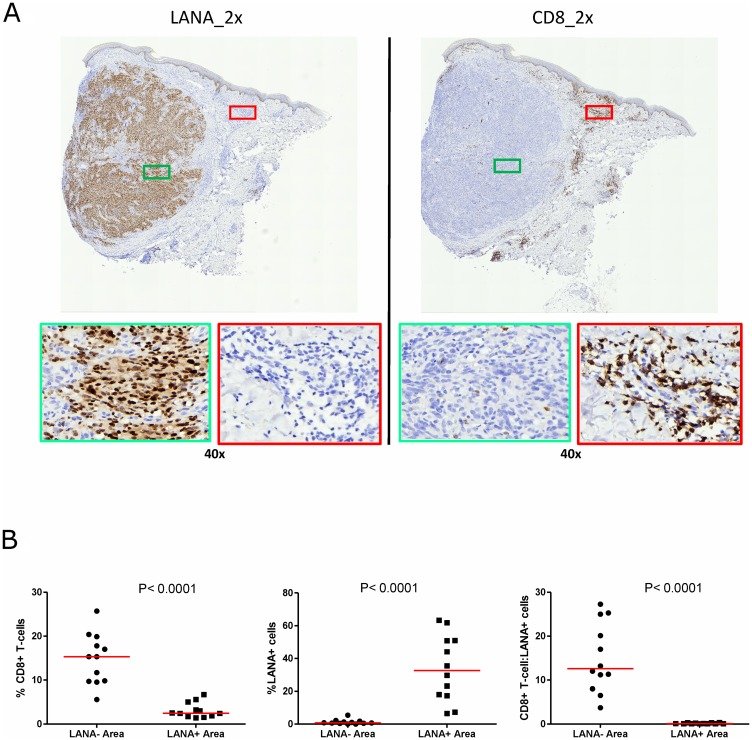
Single-color immunohistochemical staining of adjacent KS tissue sections using mouse anti-LANA and mouse anti-humanCD8 antibodies. (**A**) Representative scanned images at 2× and 40× magnification for LANA and CD8 staining ID-C3097. Green rectangle – LANA positive region of the tissue section, red rectangle – CD8^+^ T-cell region of the tissue section. (**B**) Quantification plots in LANA+ and CD8^+^ regions of the adjacent tissues sections.

Cytotoxic T-cells are anticipated to migrate into areas of foreign/tumor antigens in response to chemokines, such as CxCL-9 [27, 28]. To investigate CD8^+^ T-cell infiltration, adjacent tissues sections were IHC stained for LANA and CD8 expression, respectively. Regions with high numbers of LANA^+^ cells and high CD8^+^ T-cells on the adjacent slides were identified and statistically compared (Figure 3A). Overall, there were more CD8^+^ T-cells in KS tissue sections compared to normal skin. There was a lack of co-localization of cells with LANA signal and CD8^+^ T-cells on the adjacent section from the same tissue biopsy in 12 out of 13 analyzed KS tissues. Although CD8+ T-cells were present, but they were sparsely distributed within LANA^+^ regions of both EpKS and EnKS tissues (Figure 3A), with the majority found in regions devoid of LANA (*P* < 0.0001) (Figure 3B). These results were validated with identical findings using a different source of anti-CD8 antibody (Supplementary Figure 1).

**Figure 4:**
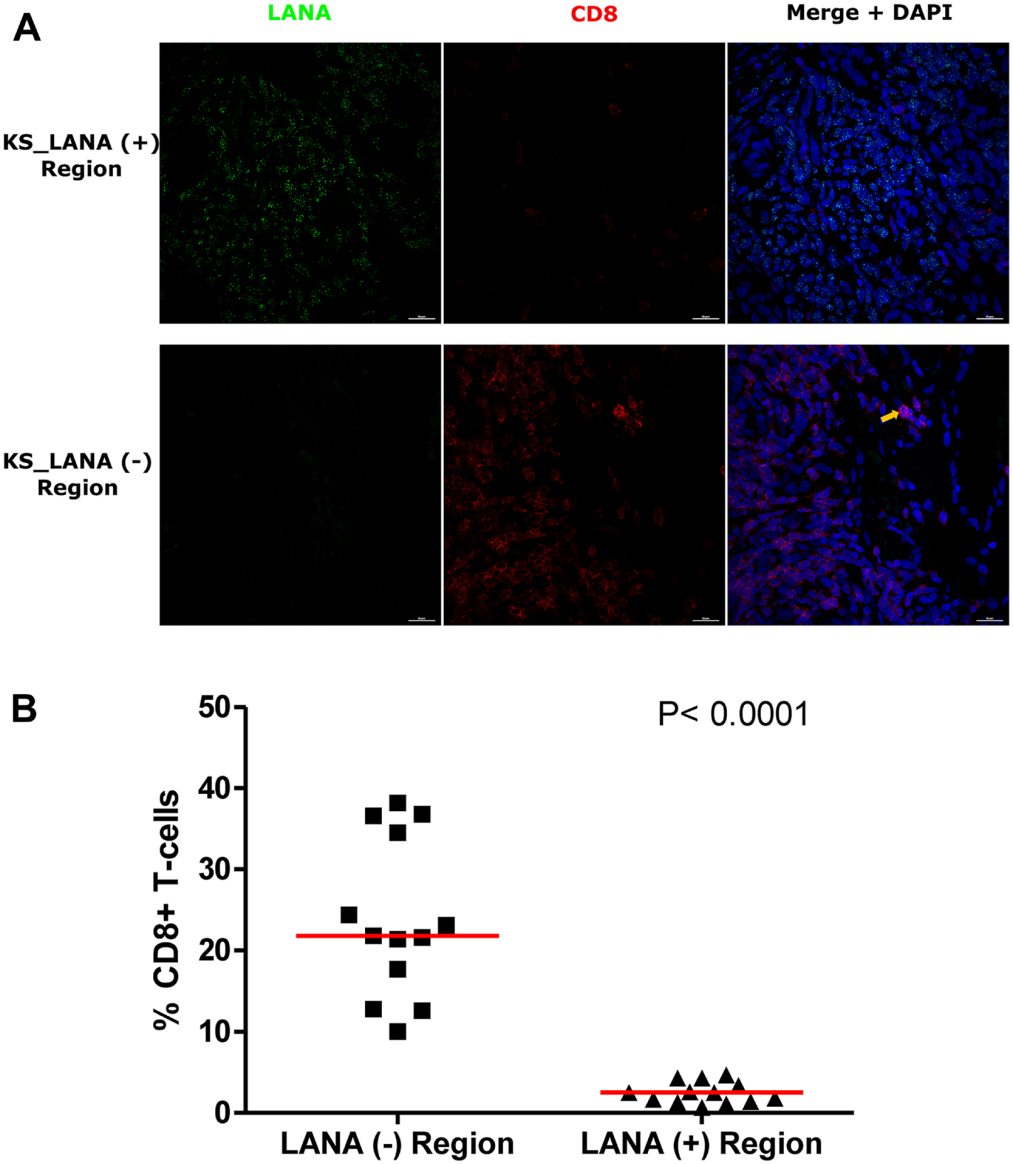
Dual-immunofluorescence staining of Kaposi’s Sarcoma-associated Herpesvirus (KSHV) Latency Associated Nuclear Antigen (LANA) protein and CD8^+^ T-cells on Kaposi’s Sarcoma (KS) tissues. (**A**) Representative images are shown ID-C038, Alexa 488 (green) for LANA, Alexa 647 (red) for CD8 and 4’,6-Diamino-2-Phenylindole, Dihydrochloride (DAPI) (blue) for nuclei staining. (**B**) Quantification plot for the percentage CD8 positive cells per field of view. Red horizontal lines indicate median. (+) – Positive and (–) – Negative. Orange arrow – CD8 positive T-cell.

The use of adjacent tissues sections with traditional single-color IHC to infer co-localization of markers is challenging, as two sequential sections need to be overlaid to determine co-localization. In order to solve this challenge and re-examine the relationship between infected cells and TIIC, we further utilized DIF to stain for LANA and CD8^+^ T-cells on the same tissue section. Consistent with the IHC results, there was a lack of co-localization of LANA^+^ cells and CD8^+^ T-cells on the same section (Figure 4A). While CD8^+^ T-cells were detectable in LANA^+^ regions of the tissues, their distribution was sparse and less abundant compared to regions devoid of LANA (*P* < 0.0001) (Figure 4B). Importantly, the proportions of CD8^+^ T-cells in LANA^+^ regions were similar to proportions in normal skin for most tumor biopsies (Supplementary Figure 2). However, CD4^+^ (Helper T-cells), CD19^+^ (B-cells) and CD56^+^ (NK cells) cells were nearly absent in most KS tissues, and when present, were at levels indistinguishable from levels in normal skin tissue (data not shown). Overall, there was lack of co-localization of CD8^+^, CD4^+^, B, or NK cells in the vicinity of KSHV infected cells.

**Figure 5:**
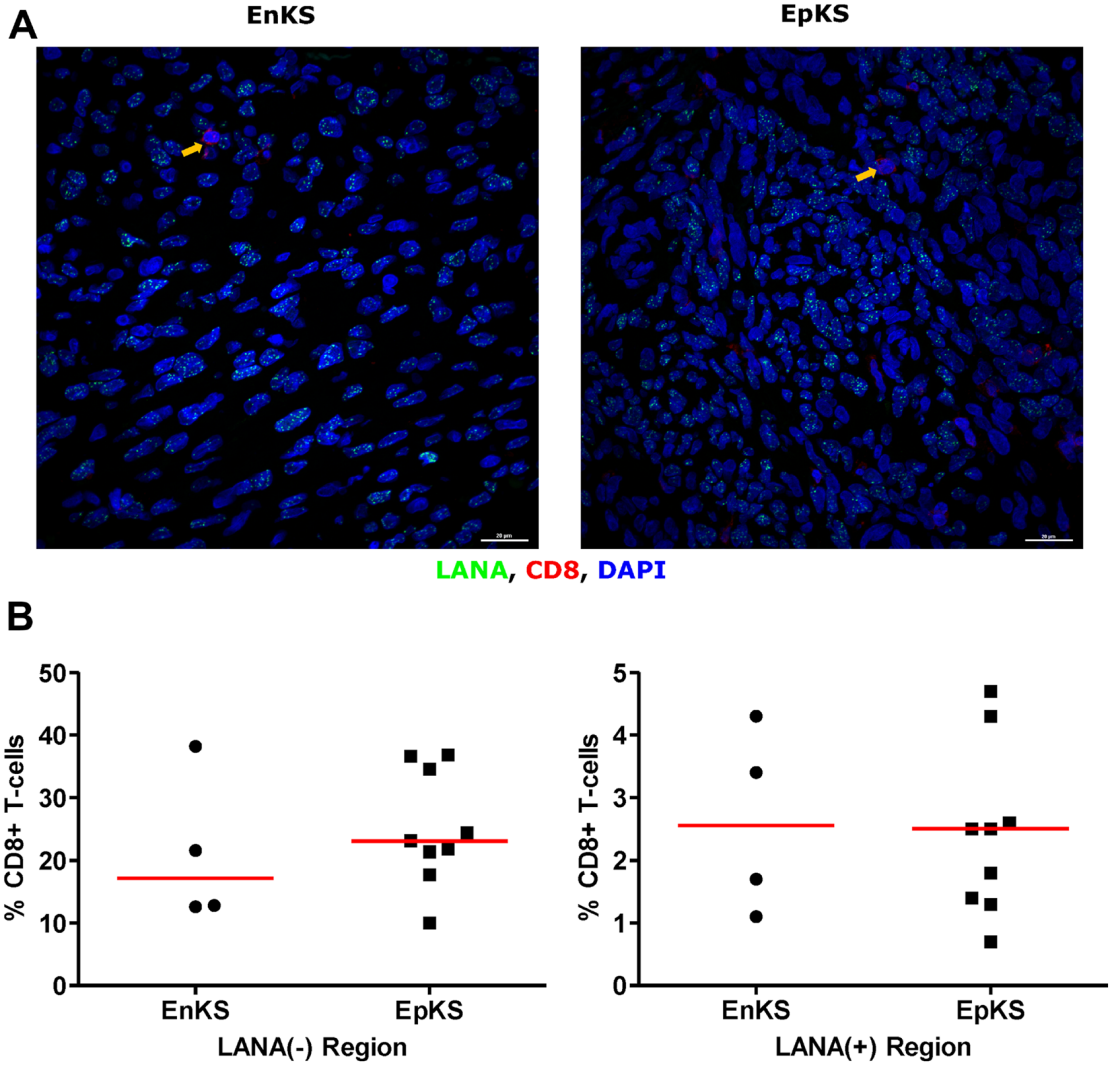
Dual-immunofluorescence staining of Kaposi’s Sarcoma-associated Herpesvirus (KSHV) Latency Associated Nuclear Antigen (LANA) protein and CD8^+^ T-cells on Kaposi’s Sarcoma (KS) tissues. (**A**) Representative images are shown EnKS – ID-C063 and EpKS – ID-C038, Alexa 488 (green) for LANA, Alexa 647 (red) for CD8 and 4’,6-Diamino-2-Phenylindole, Dihydrochloride (DAPI) (blue) for nuclei staining. (**B**) Quantification plot for the percentage CD8 positive cells per field of view. Red horizontal lines indicate median. (+) – Positive and (–) – Negative. EnKS – Endemic KS and EpKS – Epidemic KS. Orange arrows – CD8 positive T-cells.

To investigate the potential impact of HIV-1 co-infection on TIIC infiltration into KS tissues, we compared staining for CD8, CD4, B, and NK cells between EpKS and EnKS patients. The dearth of CD8^+^ T-cell infiltration into LANA^+^ regions of KS biopsies in EpKS was comparable to EnKS (Figure 5) and revealed no differences in the infiltration or co-localization of lymphocytes upon HIV-1 co-infection. Similarly, CD4 T-cells, B, and NK cells were not differential between EpKS and EnKS patients.

### CD163 expressing cells are in LANA negative region

**Figure 6:**
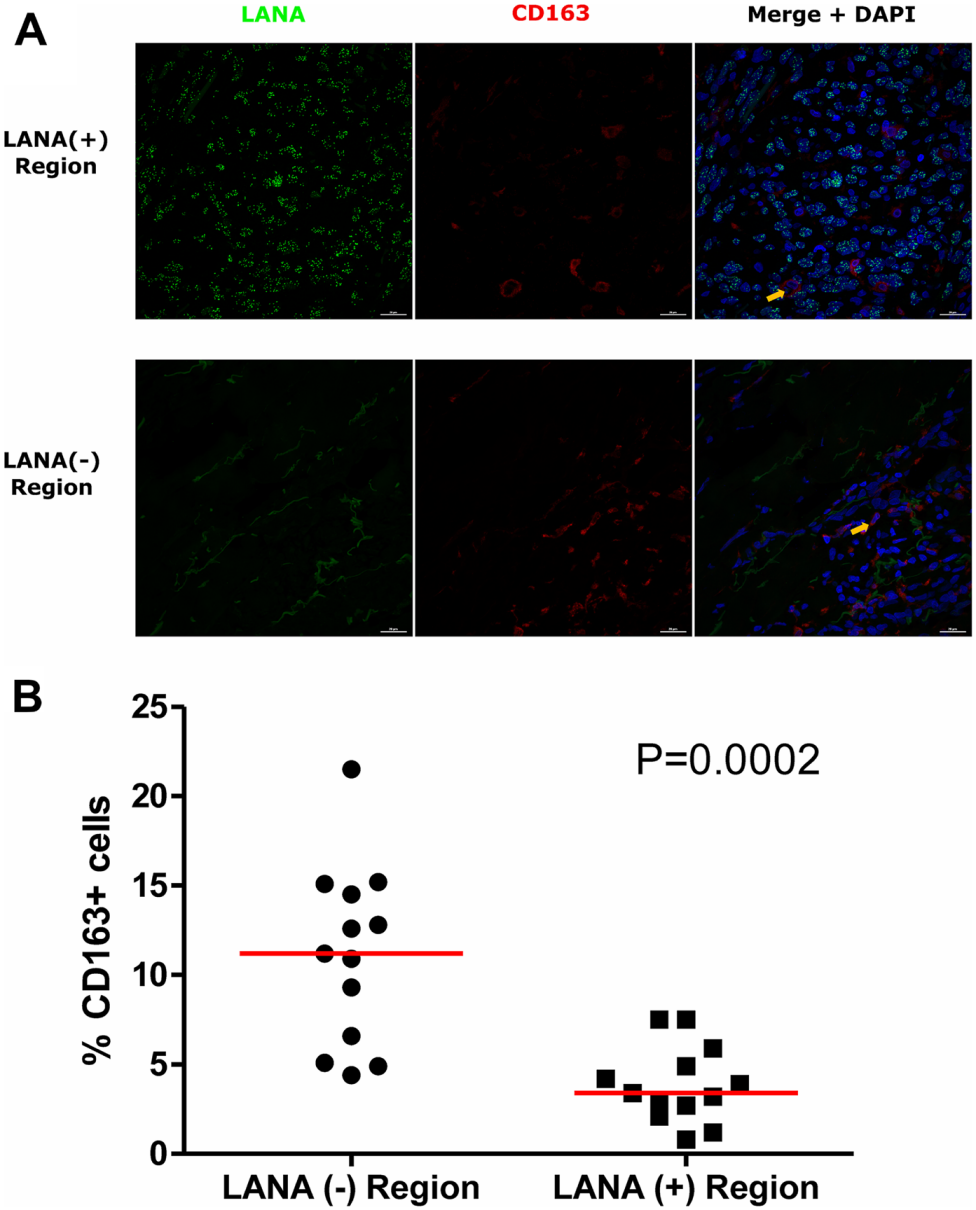
Dual-immunofluorescence staining of Kaposi’s Sarcoma-associated Herpesvirus (KSHV) Latency Associated Nuclear Antigen (LANA) protein and CD163 on Kaposi’s Sarcoma (KS) tissues. (**A**) Representative images are shown, Alexa 488 (green) for LANA, Alexa 647 (red) for CD163 and 4’,6-Diamino-2-Phenylindole, Dihydrochloride (DAPI) (blue) for nuclei staining ID-C063. (**B**) Quantification plot for the percentage CD163 positive cells per field of view. Red horizontal lines indicate median. (+) – Positive and (–) – Negative. Orange arrows – CD163 positive cells.

**Figure 7:**
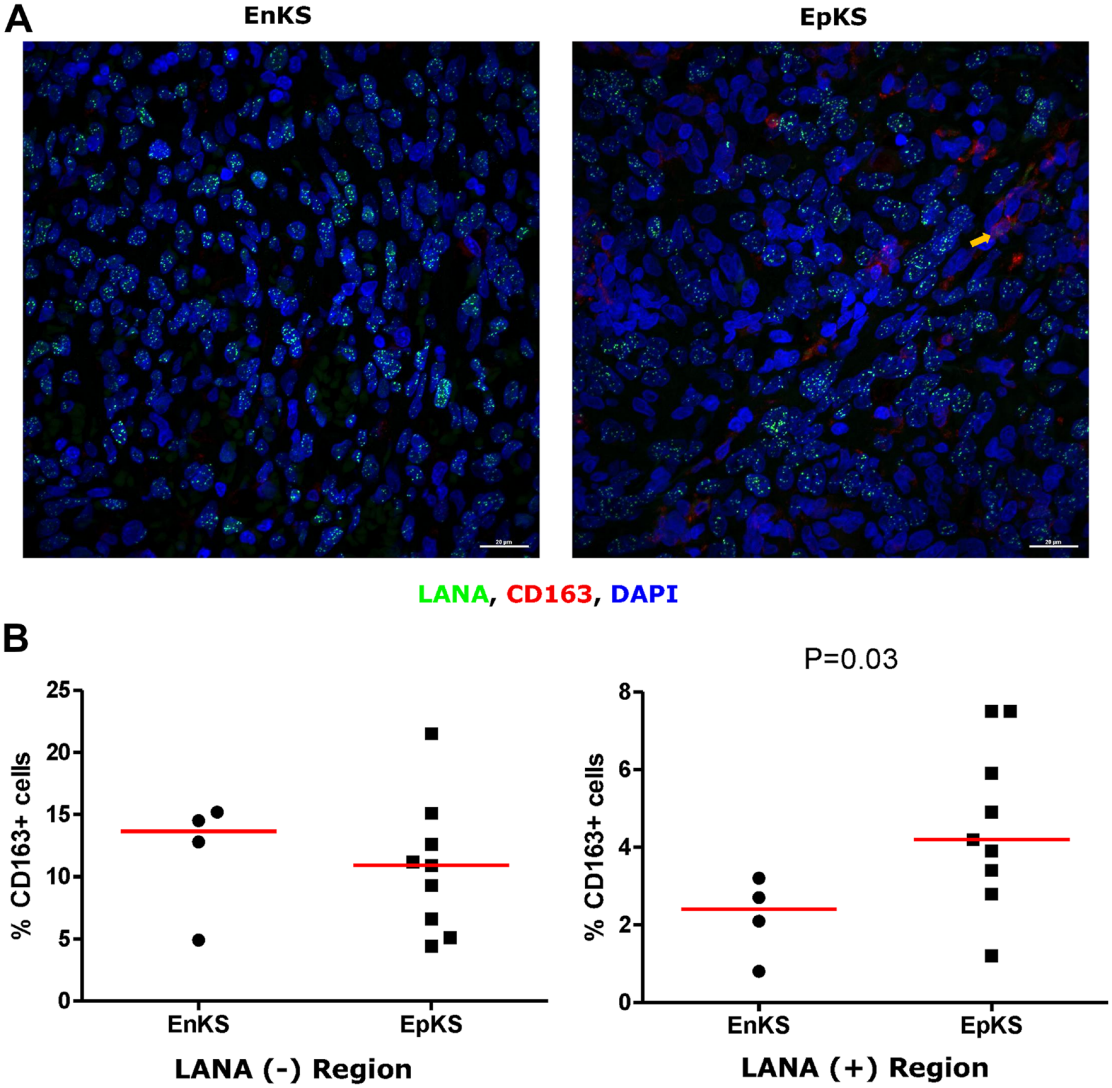
Dual-immunofluorescence staining of Kaposi’s Sarcoma-associated Herpesvirus (KSHV) Latency Associated Nuclear Antigen (LANA) protein and CD163 on Kaposi’s Sarcoma (KS) tissues. (**A**) Representative images are shown EnKS – ID-C3096 and EpKS – ID-C038, Alexa 488 (green) for LANA, Alexa 647 (red) for CD163 and 4’,6-Diamino-2-Phenylindole, Dihydrochloride (DAPI) (blue) for nuclei staining. (**B**) Quantification plot for the percentage CD163 positive cells per field of view. Red horizontal lines indicate median. (+) – Positive and (–) – Negative. EnKS – Endemic KS and EpKS – Epidemic KS. Orange arrow – CD163 positive cells.

**Figure 8:**
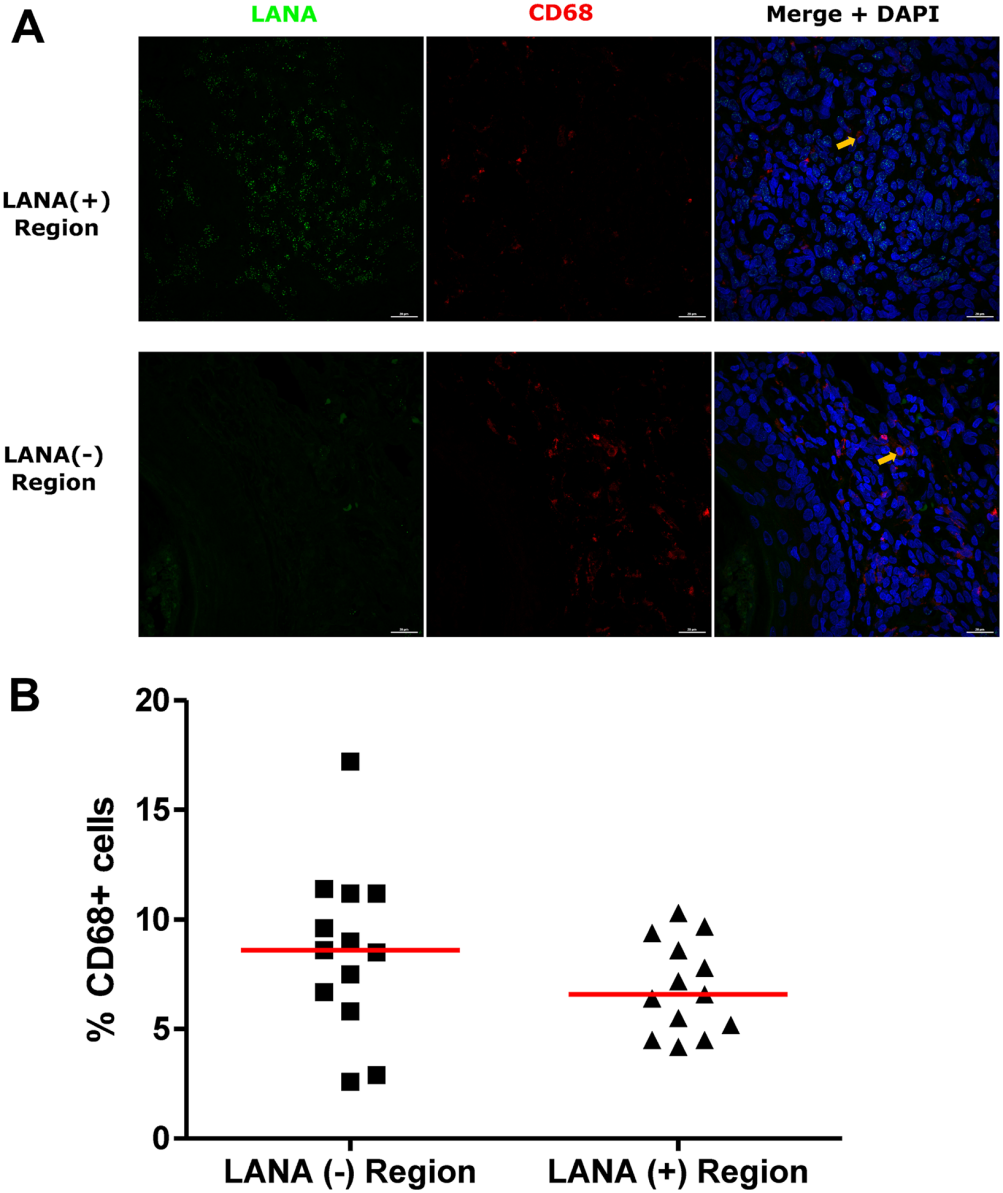
Dual-immunofluorescence staining of Kaposi’s Sarcoma-associated Herpesvirus (KSHV) Latency Associated Nuclear Antigen (LANA) protein and CD68 on Kaposi’s Sarcoma (KS) tissues. (**A**) Representative images are shown ID-C038, Alexa 488 (green) for LANA, Alexa 647 (red) for CD68 and 4’,6-Diamino-2-Phenylindole, Dihydrochloride (DAPI) (blue) for nuclei staining. (**B**) Quantification plot for the percentage CD68 positive cells per field of view. Red horizontal lines indicate median. (+) – Positive and (–) – Negative. Orange arrows – CD68 positive cells.

**Figure 9:**
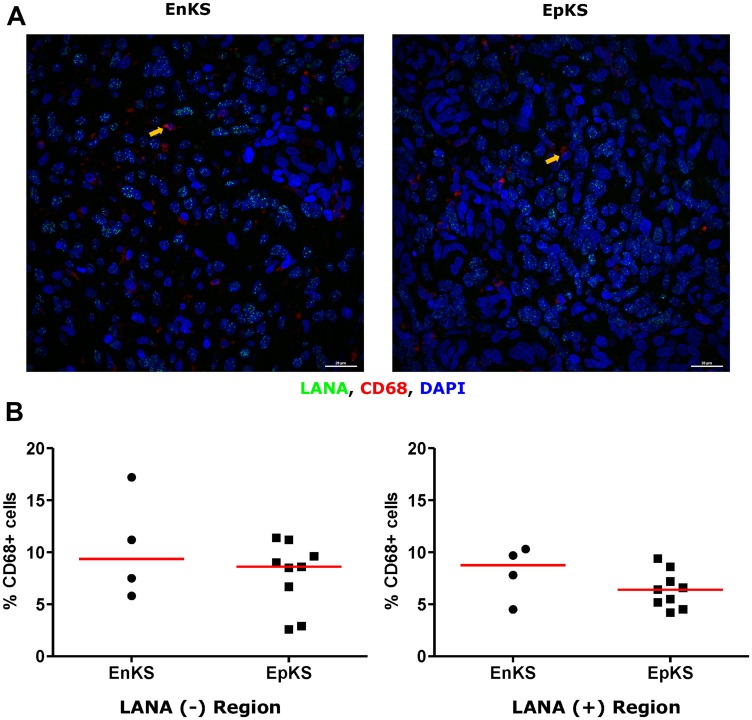
Dual-immunofluorescence staining of Kaposi’s Sarcoma-associated Herpesvirus (KSHV) Latency Associated Nuclear Antigen (LANA) protein and CD68 on Kaposi’s Sarcoma (KS) tissues. (**A**) Representative images are shown ID-C038, Alexa 488 (green) for LANA, Alexa 647 (red) for CD68 and 4’,6-Diamino-2-Phenylindole, Dihydrochloride (DAPI) (blue) for nuclei staining. (**B**) Quantification plot for the percentage CD68 positive cells per field of view. Red horizontal lines indicate median. (+) – Positive and (–) – Negative. EnKS – Endemic KS and EpKS – Epidemic KS. Orange arrows – CD68 positive cells.

**Figure 10:**
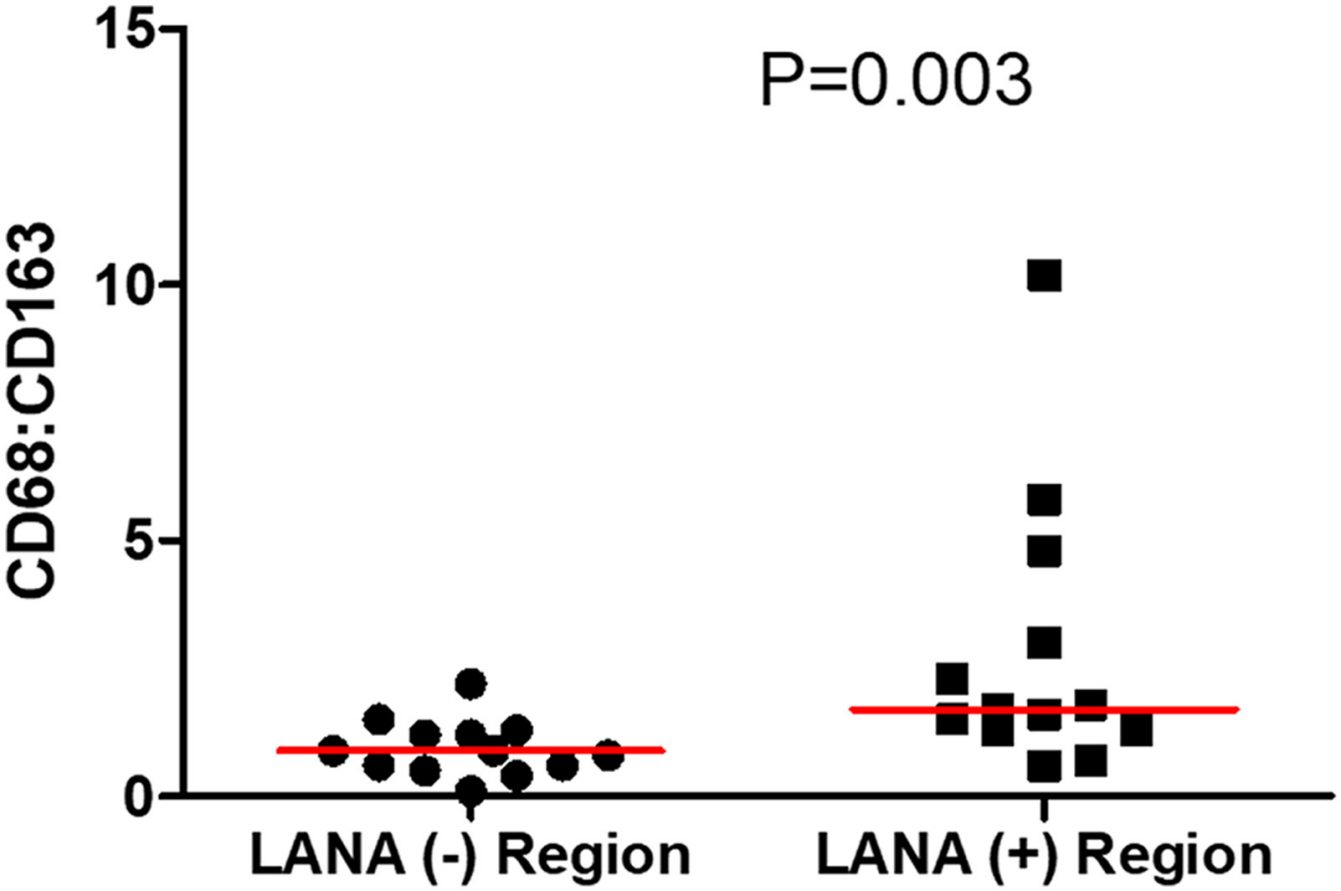
The ratio of CD68 to CD163 cells in LANA^+^ and LANA^-^ regions of KS biopsies. (+) – Positive and (–) – Negative.

Tissue resident or infiltrating macrophages can produce chemokines, including CxCL-9, to recruit T-cells to areas with antigens [29]. However, in the tumor microenvironment, macrophages are also known to produce inflammatory mediators and reactive oxygen species that can induce angiogenesis and promote tumor progression [30–32]. To investigate the M2/anti-inflammatory macrophage distribution in KS tissues, we evaluated the distribution of CD163, a hemoglobin-haptoglobin acute phase marker which is often correlated with myeloid suppressor phenotypes [33]. While CD163^+^ myeloid cells were evident in biopsied tissue, the majority were localized in LANA^-^ regions (*P* = 0.0002) (Figure 6A and 6B). Interestingly, while there were less CD163^+^ cells in LANA^+^ regions than other regions for most KS tissues, the number of CD163^+^ cells in LANA^+^ regions were significantly higher in EpKS than in EnKS (*P* = 0.03) (Figure 7A and 7B). This is consistent with increased CD163 levels of expression by the macrophages in HIV-1 infection [34, 35]. However, CD163 expression in biopsy tissue was not different between HIV-1 viremic and aviremic EpKS subjects suggesting that ART is not mitigating the impact of HIV-1 on CD163 upregulation (data not shown). We also evaluated the distribution of lectin/selectin-binding scavenger receptor, CD68 as a marker of infiltration of KS tissue by M1/inflammatory macrophages. Cells expressing CD68 were randomly distributed in KS tissues and were not significantly different between LANA^+^ or LANA^-^ regions (Figure 8). Likewise, no differences between EnKS and EpKS in CD68^+^ cell distribution was evident (Figure 9). However, the ratio of CD68 to CD163 cells was significantly higher in LANA^+^ regions compared to LANA^-^ regions of KS biopsies (Figure 10). Taken together, macrophages in KS tissues do not specifically localize with or target the KSHV infected cells. Moreover, the distribution of myeloid/macrophage cells in KS does not appear to reflect HIV-1 co-infection status or control of HIV-1 viremia.

## DISCUSSION

This is the first study to investigate spatial relationships between chemokine expression, immune cell infiltration and KSHV infected cells in EpKS and EnKS tumor tissues from SSA. Our analysis found that in both EpKS and EnKS, the abundance and distribution of LANA^+^ cells varies widely between KS biopsies even though most of the patients in this cohort had nodular KS lesions. Consistent with expression of the known T-cell chemoattractant, CxCL-9, in the KS tissues, there were more CD8^+^ T-cells in KS tissue sections compared to normal skin; however, it was notable that those T-cells did not co-localize with KSHV infected cells.

CD8^+^ T-cells play a major role in tumor and intracellular infection control through their capacity to kill infected or cancer cells in response to recognition of class I presented peptides indicative of loss of cellular homeostasis [36]. Immunosuppression induced by the tumor microenvironment can lead to dysfunctional CD8^+^ T-cells and tumor progression [37]. In solid tumors, CD8^+^ T-cell-mediated antitumor activity can be hindered by inhibitory cytokines, altered metabolism, expression of immune checkpoint molecules, abnormal tumor angiogenesis, myelosuppressive cells or regulatory T-cells (Tregs), acting alone or in combination [37, 38]. In KS, Tregs have been suggested to regulate CD8^+^ T-cells [39]. To evaluate which of these potential mechanisms might be contributory to KS pathogenesis, we have initially evaluated markers and cell types that were found to be elevated in our KS transcriptomics analysis that compared KS tissue to contralateral skin from the same subjects [17]. That study suggested over-expression of T-cell chemoattractant genes and those indicative of immune cell infiltration coupled to a Warburg-like metabolic pattern. Quantification of the extent of CD8^+^ T-cell infiltration into KS tissues revealed a distribution that was limited mainly to areas devoid of KSHV LANA^+^ cells. This was surprising, since the T-cell chemoattractant, CxCL-9, was demonstrated to be elevated at the protein level in KS tissues sections compared to normal skin. Importantly, other T-cell chemoattractants, CxCL-10 and 11 transcripts were also overexpressed in KS lesions compared to controls [17], but we have not yet attempted detection of those proteins in tissue. While CD8^+^ T-cells were clearly present in the vicinity of KS tumor tissue in excess of the numbers in normal skin, it is possible that recruited CD8^+^ T-cells undergo inhibition from exposure to the cytokines produced by the tumor cells like IL-10 and TGF-β, that may impede access to the tumor parenchyma [17, 38].

Like other herpesviruses, KSHV is known to downregulate MHC class I antigen presentation and T-cell co-stimulation molecules CD86 and ICAM [40, 41]. Through viral encoded K3 and K5, KSHV escapes detection by cytotoxic T-cells and NK cells [42, 43]. vIRF3 also downregulates MHC class II by degrading interferon-γ produced by NK cells [44]. It is possible that the lack of T-cell co-localization with KSHV infected cells in KS tumors is a result of KSHV downregulation of interferon responses and antigen presentation. The lack of co-localization could also be due to the lack of T-cell repertoire against KSHV in the T-cells that chemotactically locate the infected cells in the KS tumor. Studies of circulating T-cell Ag-specific function suggest lack of immunodominance against KSHV antigens in either KS or asymptomatic patients. Although some T-cell repertoire against KSHV has been reported, most of these responses were weak and variable in different individuals [45, 46]. It is also possible that the viral peptides presented by Class I on the KS tumors are decoupled from T-cell repertoire recognition, thereby shielding the antigens from the immune systems. This is currently under investigation.

KSHV is known to induce hypoxic metabolic derangements through HIF-1 expression [47]. The induced metabolic dysfunctions, including Warburg-like metabolism, could result in lactate production. The resultant hypoxic and acidic tumor environment could be non-conducive for T-cell infiltration leading to restriction of CD8^+^ T-cells access. Although, it appears unlikely given the progressive nature of KS disease, it is also possible that LANA^+^ cells were previously targeted and killed by CD8^+^ T-cells in regions exhibiting enriched T-cells but devoid of LANA^+^ cells. However, T-cells in these focal areas would have to have become exhausted and unable to proliferate and infiltrate the rest of the tumor [48]. Further studies will be needed to address the immunophenotypes, functionality and KSHV specificity of the CD8^+^ T-cells and other immune cells from the immune cell infiltrates in KS biopsies. Analysis of MHC class I peptides presented on KS tumors is also important in order to rule out mismatch between T-cell repertoire and Ag presentation repertoire.

Macrophages detect, engulf and destroy invading microorganisms and infected cells [31, 49]. During tumor progression, tumor cells induce maladaptive macrophage phenotypes through metabolic reprogramming [50]. The maladapted macrophages create an immunosuppressive niche leading to tumor progression and therapeutic resistance [51]. Cytokines such as IL-6, IL-10 and TGF-β are known to polarize macrophages into the M2 phenotype that is associated with immunosuppression [9, 30, 52]. Interestingly our previous study has shown that IL-6, IL-10, IL-5 and TGF-β were elevated in KS patients compared to asymptomatic controls [5]. These cytokines were also associated with increased antibody responses that are not protective against KS [5]. High levels of macrophages in KS biopsies could be responsible for creating an immunosuppressive niche that restricts CD8+ T-cell infiltration and enhance tumor growth. Overall, it appears that macrophages in KS tumors support KS progression. However, investigation of different macrophage phenotypes and their specific functions in KS biopsies is needed to understand the role of TIIC in KS.

This study initiated the investigation of TIIC in KS biopsies in order to understand the role of TIIC in KS. Overall, there is poor immune cell infiltration or co-localization in LANA^+^ regions of KS biopsies. The apparent lack of immune cell co-localization with KSHV infected cells for both EpKS and EnKS suggests that HIV-1 does not significantly influence immune cell infiltration in KS biopsies. However, the size of the cohort, the formalin-fixed treatment of the samples as well as the limited tumor and control tissue quantity, have reduced the depth of our investigations. Studies with larger sample sizes patients will be needed to address gaps highlighted in this study. Whether TIICs in KS are functional and match presented peptides in the tumor also need to be further investigated with freshly collected tissue. Correlation between TIIC abundance and treatment outcomes of KS could be useful in defining the role of TIIC as a prognostic marker of KS.

## MATERIALS AND METHODS

### Study design, subjects and samples

This cross-sectional study recruited 16 participants (4 EnKS, 9 EpKS KS patients and 3 controls without KS) from Tanzania and Zambia. Participants were of both genders and over 18 years old. Written informed consent was obtained from all study participants. KS was diagnosed by both histology and immunohistochemistry for KSHV latency associated nuclear antigen (LANA). Peripheral blood samples about 10 mL were collected, plasma was isolated and used for HIV-1 viral load quantification by real-time PCR. A 4 mm KS biopsy was taken from each subject, fixed in neutral buffered formalin, and processed into a paraffin-embedded block for KS histological diagnosis, LANA IHC and subsequent immunofluorescence staining. This study was approved by the review boards of Tanzania National Institute for Medical Research, Ocean Road Cancer Institute, University of Zambia Biomedical Research Ethics Committee and the University of Nebraska-Lincoln (UNL).

### HIV-1 serology and plasma viral load quantification by real-time PCR (RT-PCR)

HIV-1 serology was determined by point-of-care serology and plasma viral load by quantitative RT-PCR as previously described [5]. Briefly, HIV-1 screening was done using HIV Rapid Test Algorithm in Tanzania or Alere Determine HIV-1/2 Ag/Ab Combo test in Zambia [53]. Viral RNA was extracted from plasma according to the QIAamp viral RNA extraction protocol (Qiagen, Hilden, Germany). The viral copy numbers were then determined using RNA Ultra-Sense One-Step quantitative RT-PCR system (Applied Biosystems, Carlsbad, CA) as previously described with universal HIV LTR primers [54] (forward [5′-GCCTCAATAAAGCTTGCCTTGA-3′] and reverse [5′–GGGCGCCACTGCTAGAGA–3′] and probe [5′-FAM/CCAGAGTCACACAACAGACGGGCACA/-BHQ1-3′]) under the following conditions: 50°C for 15 min, 95°C for 2 min, 40 cycles of 95°C for 15 seconds, and 60°C for 30 seconds.

### Immunohistochemistry (IHC) and cell quantification

Adjacent 6 μm-thick sections of paraffin-embedded KS tissues were used for IHC, as previously described [55]. Mouse anti-LANA antibody clone NCL-L-HHV8-LNA (1:100) (Leica Biosystems, Newcastle, UK), anti-human CD8 clone C8/144B (1:100) (Agilent, Santa Clara, CA) or anti-human CD8 clone 4B11 (1:40) (GeneTex, Irvine, CA) were used as primary antibodies. Normal mouse IgG (Dako ×0931, Agilent, Santa Clara, CA) was used as an isotype control at identical concentration.

To quantify the number of positive cells for a specific cell surface marker of interest, all IHC stained slides were digitally scanned on a MoticEasyScan One (Motic, San Antonio, TX) at 40 ×. On each anti-LANA stained slide, five regions of about 50 μm^2^ were selected in regions with high abundance of LANA. The total number of cells and LANA^+^ cells were then quantified using Fiji software (NIH). The same procedure was applied to anti-CD8 stained slides and CD8+ cells were counted. The five high LANA regions were then identified on corresponding CD8 stained slides and the total number of cells and CD8^+^ T-cells were counted. Reciprocally, the five high CD8+ regions were then identified on corresponding LANA stained slides and the total number of cells and LANA+ cells were counted. Average number of total cells and marker-delineated cells in the five regions were used to calculate proportions of specific markers in an area of high LANA or high CD8^+^ T-cells on adjacent tissue sections. Percentages of specific markers were then calculated and plotted using GraphPad Prism v.5 (GraphPad Software, San Diego, CA).

### Dual-Color Immunofluorescence (DIF)

Adjacent 6 μm-thick sections of paraffin-embedded KS tissues were processed following the IHC protocol with modifications. After antigen retrieval, the sections were incubated with 0.3M glycine for 30 min at room temperature to minimize tissue auto-fluorescence and Tris-NaCl blocking buffer (PerkinElmer FP1020, Boston, MA) was used as blocking solution. For DIF, a rat anti-LANA antibody clone LN35 at 1:100 (Abcam, Cambridge, MA) was used together with one of the following primary mouse monoclonal antibodies: anti-CD8 clone C8/144B (1:100), anti-CD4 clone 4B12 (1:100), anti CD34 clone QBEnd10 (1:50), anti-CD68 clone KP1 (1:100), CD19 clone LE-CD19 and anti-CD56 clone 123C3 (1:100) (Agilent, Santa Clara, CA) and anti-CD163 clone10D6 (1:100) (Novus Biologicals, Centennial, CO) or goat polyclonal anti-CxCL-9/MIG clone AF392 (R&D systems, Minneapolis, MN). The primary antibodies were diluted in TNB blocking buffer and incubated at 4°C overnight in a humidity chamber. Sections were equilibrated to room temperature, rinsed in 1× PBS-0.05% Tween-20 and then incubated with secondary antibodies Alexa 488-Donkey anti-rat A21208 together with either Alexa 647-Donkey anti-mouse A31571 (Invitrogen, Eugene, OR) or Alexa 647-Donkey anti-goat A32849, Invitrogen, Rockford IL) for 2 hours at room temperature in a humidity chamber. Nucleus counterstaining was performed with 300 nM 4’,6-Diamino-2-Phenylindole, Dihydrochloride (DAPI) solution (Invitrogen D1306, Eugene OR) for 30 minutes before mounting coverslip with Fluoro-gel (# 17985-10, Electron Microscopy Sciences, Hatfield PA). To determine lineage of the KSHV infected cells in the KS tumors, endothelial cell marker CD34 was utilized, consistent with other literature on KS where CD34 has been utilized to demonstrate endothelial lineage [22, 23].

### Confocal microscopy and cell quantification

All sections were imaged with Nikon A1R laser scanning confocal system mounted on Nikon Ti2 inverted fluorescence microscope (Nikon Instruments, Melville, NY). Nuclei stained with DAPI were excited at 405 nm wavelength and emission was collected between 425–475 nm wavelengths. Alexa 488 stained LANA was excited at 488 nm wavelength and emission was collected between 500–550nm wavelengths. All other surface markers were stained with Alexa 647, and excited at 640 nm with emission collection between 663–738 nm wavelengths. Images were acquired with NIS elements software (Nikon Instruments, Melville, NY) using sequential scanning (channel series) and Z-stacking (Z-series) of 1 μm slices at 60× magnification. Settings for laser power, detector gain and offset for individual markers were unchanged between slides. LANA positive (+) and negative (–) regions were identified in the same tissue and three representative regions with maximum signal (LANA and surface marker) were selected and imaged. Fiji software (NIH) was used on maximum image projections (MaxIP) to quantify the total number of cells, and cells with specific marker enumeration. The average of total number of cells and cells with specific markers in the three regions was used to calculate proportions of specific markers in an area of high LANA and in a region with high CD8^+^ T-cells. Percentages of specific markers were then calculated and plotted using GraphPad Prism v.5 (GraphPad Software, San Diego, CA).

### Statistical analysis

Cell surface marker expression was compared between KS tissues and normal skin, between KSHV LANA (+) and (–) regions of the same section or between adjacent sections and between EpKS and EnKS patients. At least 3 sections per tissue were evaluated for all 16 tissue samples. 3 to 5 regions per tissue section were quantified and the average number of cells was used for statistical analysis. All statistical analyses were performed by analysis of variance (ANOVA) and non-parametric Mann-Whitney test using GraphPad Prism v.5 (GraphPad Software, San Diego, CA). All tests were 2-tailed, and *P*-values < 0.05 were considered significant.

## SUPPLEMENTARY MATERIALS



## Abbreviations

KS: Kaposi’s sarcoma
EpKS: Epidemic Kaposi’s sarcoma
EnKS: Endemic Kaposi’s sarcoma
KSHV: Kaposi’s sarcoma associated herpesvirus
TIIC: Tumor infiltrating immune cells
ART: Antiretroviral therapy
HIV-1: Human Immunodeficiency virus type 1
AIDS: Acquired Immunodeficiency syndrome
CxCL-9: Chemokine (CxC motif) ligand 9
LANA: Latency associated nuclear antigen
Treg: Regulatory T-cell
HIV-1^-^: HIV-1 negative
HIV-1^+^: HIV-1 positive
IHC: Immunohistochemistry
DIF: dual-color immunofluorescence
IL-10: Interleukin-10
IL-6: Interleukin-6
IL-5: Interleukin-5
TGF-β: Transforming growth factor β
DAPI: 4’, 6-Diamino-2-Phenylindole, Dihydrochloride
PCR: Polymerase chain reaction
SSA: sub-Saharan Africa

## References

[1] Duman S . Successful treatment of post-transplant Kaposi’s sarcoma by reduction of immunosuppression. Nephrol Dial Transplant. 2002; 17:892–6. 10.1093/ndt/17.5.892. 11981080

[2] Franceschi S , Maso LD , Rickenbach M , Polesel J , Hirschel B , Cavassini M , Bordoni A , Elzi L , Ess S , Jundt G , Mueller N , Clifford GM , Battegay M , et al. Kaposi sarcoma incidence in the Swiss HIV Cohort Study before and after highly active antiretroviral therapy. Br J Cancer. 2008; 99:800–804. 10.1038/sj.bjc.6604520. 18665172 PMC2528138

[3] Cook-Mozaffari P , Newton R , Beral V , Burkitt DP . The geographical distribution of Kaposi’s sarcoma and of lymphomas in Africa before the AIDS epidemic. Br J Cancer. 1998; 78:1521–1528. 10.1038/bjc.1998.717. 9836488 PMC2063225

[4] Labo N , Marshall V , Miley W , Davis E , McCann B , Stolka KB , Ndom P , Hemingway-Foday JJ , Abassora M , Newton R , Smith JS , Whitby D . Mutual detection of Kaposi’s sarcoma-associated herpesvirus and Epstein–Barr virus in blood and saliva of Cameroonians with and without Kaposi’s sarcoma. Int J Cancer. 2019; 145:2468–77. 10.1002/ijc.32546. 31265124

[5] Lidenge SJ , Tso FY , Ngalamika O , Ngowi JR , Mortazavi Y , Kwon EH , Shea DM , Minhas V , Mwaiselage J , Wood C , West JT . Similar immunological profiles between African endemic and human immunodeficiency virus type 1-associated epidemic Kaposi Sarcoma (KS) patients reveal the primary role of KS-associated herpesvirus in KS pathogenesis. J Infect Dis. 2019; 219:1318–28. 10.1093/infdis/jiy654. 30452681 PMC6452303

[6] Odunsi K , Old LJ . Tumor infiltrating lymphocytes: indicators of tumor-related immune responses. Cancer Immun. 2007; 7:3. 17311362 PMC2935754

[7] Chen J , He Q , Liu J , Xiao Y , Xiao C , Chen K , Xie D , Zhang X . CD8+ tumor-infiltrating lymphocytes as a novel prognostic biomarker in lung sarcomatoid carcinoma, a rare subtype of lung cancer. Cancer Manag Res. 2018; 10:3505–3511. 10.2147/CMAR.S169074. 30271199 PMC6145683

[8] Fu Q , Chen N , Ge C , Li R , Li Z , Zeng B , Li C , Wang Y , Xue Y , Song X , Li H , Li G . Prognostic value of tumor-infiltrating lymphocytes in melanoma: a systematic review and meta-analysis. Oncoimmunology. 2019; 8:1593806. 10.1080/2162402X.2019.1593806. 31143514 PMC6527267

[9] Barros MH , Hauck F , Dreyer JH , Kempkes B , Niedobitek G . Macrophage Polarisation: an Immunohistochemical Approach for Identifying M1 and M2 Macrophages. PLoS One. 2013; 8:e80908. 10.1371/journal.pone.0080908. 24260507 PMC3829941

[10] Yang L , Wang F , Wang L , Huang L , Wang J , Zhang B , Zhang Y . CD163+ tumor-associated macrophage is a prognostic biomarker and is associated with therapeutic effect on malignant pleural effusion of lung cancer patients. Oncotarget. 2015; 6:10592–603. 10.18632/oncotarget.3547. 25871392 PMC4496378

[11] Kubota K , Moriyama M , Furukawa S , Rafiul HASM , Maruse Y , Jinno T , Tanaka A , Ohta M , Ishiguro N , Yamauchi M , Sakamoto M , Maehara T , Hayashida JN , et al. CD163+CD204+ tumor-associated macrophages contribute to T cell regulation via interleukin-10 and PD-L1 production in oral squamous cell carcinoma. Sci Rep. 2017; 7:1755. 10.1038/s41598-017-01661-z. 28496107 PMC5431876

[12] Mills CD , Lenz LL , Harris RA . A breakthrough: Macrophage-directed cancer immunotherapy. Cancer Research. 2016; 76:513–516. 10.1158/0008-5472.CAN-15-1737. 26772756 PMC4738030

[13] Stevanović S , Draper LM , Langhan MM , Campbell TE , Kwong ML , Wunderlich JR , Dudley ME , Yang JC , Sherry RM , Kammula US , Restifo NP , Rosenberg SA , Hinrichs CS . Complete regression of metastatic cervical cancer after treatment with human papillomavirus-targeted tumor-infiltrating T cells. J Clin Oncol. 2015; 33:1543–1550. 10.1200/JCO.2014.58.9093. 25823737 PMC4417725

[14] Mullinax JE , Hall M , Prabhakaran S , Weber J , Khushalani N , Eroglu Z , Brohl AS , Markowitz J , Royster E , Richards A , Stark V , Zager JS , Kelley L , et al. Combination of ipilimumab and adoptive cell therapy with tumor-infiltrating lymphocytes for patients with metastatic melanoma. Front Oncol. 2018; 8:44. 10.3389/fonc.2018.00044. 29552542 PMC5840208

[15] Yao W , He J , Yang Y , Wang J , Qian Y , Yang T , Ji L . The Prognostic Value of Tumor-infiltrating Lymphocytes in Hepatocellular Carcinoma: a Systematic Review and Meta-analysis. Sci Rep. 2017; 7:7525. 10.1038/s41598-017-08128-1. 28790445 PMC5548736

[16] Antohe M , Nedelcu R , Nichita L , Popp C , Cioplea M , Brinzea A , Hodorogea A , Calinescu A , Balaban M , Ion D , Diaconu C , Bleotu C , Pirici D , et al. Tumor infiltrating lymphocytes: The regulator of melanoma evolution (Review). Oncol Lett. 2019; 17:4155–61. 10.3892/ol.2019.9940. 30944610 PMC6444298

[17] Tso FY , Kossenkov AV , Lidenge SJ , Ngalamika O , Ngowi JR , Mwaiselage J , Wickramasinghe J , Kwon EH , West JT , Lieberman PM , Wood C . RNA-Seq of Kaposi’s sarcoma reveals alterations in glucose and lipid metabolism. PLoS Pathog. 2018; 14:e1006844. 10.1371/journal.ppat.1006844. 29352292 PMC5792027

[18] Bromley SK , Mempel TR , Luster AD . Orchestrating the orchestrators: Chemokines in control of T cell traffic. Nat Immunol. 2008; 9:970–980. 10.1038/ni.f.213. 18711434

[19] Chalya PL , Mbunda F , Rambau PF , Jaka H , Masalu N , Mirambo M , Mushi MF , Kalluvya SE . Kaposi’s sarcoma: a 10-year experience with 248 patients at a single tertiary care hospital in Tanzania. BMC Res Notes. 2015; 8:440. 10.1186/s13104-015-1348-9. 26374100 PMC5439227

[20] Garrigues HJ , Howard K , Barcy S , Ikoma M , Moses AV , Deutsch GH , Wu D , Ueda K , Rose TM . Full-Length Isoforms of Kaposi’s Sarcoma-Associated Herpesvirus Latency-Associated Nuclear Antigen Accumulate in the Cytoplasm of Cells Undergoing the Lytic Cycle of Replication. J Virol. 2017; 91. 10.1128/jvi.01532-17. 28978712 PMC5709576

[21] Uppal T , Banerjee S , Sun Z , Verma SC , Robertson ES . KSHV LANA—The Master Regulator of KSHV Latency. Viruses. 2014; 6:4691–4998. 10.3390/v6124961. 25514370 PMC4276939

[22] Cancian L , Hansen A , Boshoff C . Cellular origin of Kaposi’s sarcoma and Kaposi’s sarcoma-associated herpesvirus-induced cell reprogramming. Trends Cell Biol. 2013; 23:421–32. 10.1016/j.tcb.2013.04.001. 23685018

[23] Li Y , Zhong C , Liu D , Yu W , Chen W , Wang Y , Shi S , Yuan Y . Evidence For Kaposi’s Sarcoma Originating From Mesenchymal Stem Cell Through KSHV-induced Mesenchymal-to-Endothelial Transition. Cancer Res. 2018; 78:230–245. 10.1158/0008-5472.CAN-17-1961. 29066510 PMC5754241

[24] Muniraju M , Mutsvunguma LZ , Foley J , Escalante GM , Rodriguez E , Nabiee R , Totonchy J , Mulama DH , Nyagol J , Wussow F , Barasa AK , Brehm M , Ogembo JG . Kaposi Sarcoma-Associated Herpesvirus Glycoprotein H Is Indispensable for Infection of Epithelial, Endothelial, and Fibroblast Cell Types. J Virol. 2019; 93. 10.1128/jvi.00630-19. 31142670 PMC6675886

[25] Rappocciolo G , Jais M , Piazza PA , DeLucia DC , Jenkins FJ , Rinaldo CR . Human Herpesvirus 8 Infects and Replicates in Langerhans Cells and Interstitial Dermal Dendritic Cells and Impairs Their Function. J Virol. 2017; 91. 10.1128/jvi.00909-17. 28768873 PMC5625489

[26] Faure A , Hayes M , Sugden B . How Kaposi’s sarcoma-associated herpesvirus stably transforms peripheral B cells towards lymphomagenesis. Proc Natl Acad Sci U S A. 2019; 116:16519–28. 10.1073/pnas.1905025116. 31363046 PMC6697783

[27] Ochiai E , Sa Q , Brogli M , Kudo T , Wang X , Dubey JP , Suzuki Y . CXCL9 is important for recruiting immune T cells into the brain and inducing an accumulation of the T cells to the areas of tachyzoite proliferation to prevent reactivation of chronic cerebral infection with Toxoplasma gondii. Am J Pathol. 2015; 185:314–24. 10.1016/j.ajpath.2014.10.003. 25432064 PMC4305179

[28] Tokunaga R , Zhang W , Naseem M , Puccini A , Berger MD , Soni S , McSkane M , Baba H , Lenz HJ . CXCL9, CXCL10, CXCL11/CXCR3 axis for immune activation – A target for novel cancer therapy. Cancer Treatment Reviews. 2018; 63:40–47. 10.1016/j.ctrv.2017.11.007. 29207310 PMC5801162

[29] Arango Duque G , Descoteaux A . Macrophage cytokines: involvement in immunity and infectious diseases. Front Immunol. 2014; 5:491. 10.3389/fimmu.2014.00491. 25339958 PMC4188125

[30] Nielsen SR , Schmid MC . Macrophages as Key Drivers of Cancer Progression and Metastasis. Mediators of Inflammation. 2017; 2017:9624760. 10.1155/2017/9624760. 28210073 PMC5292164

[31] Hu K , Jin Y , Chroneos Z , Han X , Liu H , Lin L . Macrophage functions and regulation: Roles in diseases and implications in therapeutics. Journal of Immunology Research. 2018; 2018:7590350. 10.1155/2018/7590350. 29967802 PMC6008777

[32] El-Kenawi A . With macrophages, tumors won’t go hungry. Sci Transl Med. 2019; 11:eaax1722 10.1126/scitranslmed.aax1722.

[33] Cheng Z , Zhang D , Gong B , Wang P , Liu F . CD163 as a novel target gene of STAT3 is a potential therapeutic target for gastric cancer. Oncotarget. 2017; 8:87244–87262. 10.18632/oncotarget.20244. 29152078 PMC5675630

[34] Tippett E , Cheng WJ , Westhorpe C , Cameron PU , Brew BJ , Lewin SR , Jaworowski A , Crowe SM . Differential Expression of CD163 on Monocyte Subsets in Healthy and HIV-1 Infected Individuals. PLoS One. 2011; 6:e19968. 10.1371/journal.pone.0019968. 21625498 PMC3098854

[35] D’Antoni ML , Byron MM , Chan P , Sailasuta N , Sacdalan C , Sithinamsuwan P , Tipsuk S , Pinyakorn S , Kroon E , Slike BM , Krebs SJ , Khadka VS , Chalermchai T , et al. Normalization of Soluble CD163 Levels After Institution of Antiretroviral Therapy During Acute HIV Infection Tracks with Fewer Neurological Abnormalities. J Infect Dis. 2018; 218:1453–63. 10.1093/infdis/jiy337. 29868826 PMC6151077

[36] Durgeau A , Virk Y , Corgnac S , Mami-Chouaib F . Recent advances in targeting CD8 T-cell immunity for more effective cancer immunotherapy. Frontiers in Immunology. 2018; 9:14. 10.3389/fimmu.2018.00014. 29403496 PMC5786548

[37] Farhood B , Najafi M , Mortezaee K . CD8+ cytotoxic T lymphocytes in cancer immunotherapy: A review. Journal of Cellular Physiology. 2019; 234:8509–8521. 10.1002/jcp.27782. 30520029

[38] Maimela NR , Liu S , Zhang Y . Fates of CD8+ T cells in Tumor Microenvironment. Computational and Structural Biotechnology Journal. 2019; 17:1–13. 10.1016/j.csbj.2018.11.004. 30581539 PMC6297055

[39] Lepone LM , Rappocciolo G , Piazza PA , Campbell DM , Jenkins FJ , Rinaldo CR . Regulatory T Cell Effect on CD8 + T Cell Responses to Human Herpesvirus 8 Infection and Development of Kaposi’s Sarcoma. AIDS Res Hum Retroviruses. 2017; 33:668–674. 10.1089/aid.2016.0155. 28121161 PMC5512339

[40] Coscoy L , Sanchez DJ , Ganem D . A novel class of herpesvirus-encoded membrane-bound E3 ubiquitin ligases regulates endocytosis of proteins involved in immune recognition. J Cell Biol. 2001; 155:1265–73. 10.1083/jcb.200111010. 11756476 PMC2199318

[41] Lehner PJ , Hoer S , Dodd RB , Duncan LM . Downregulation of cell surface receptors by the K3 family of viral and cellular ubiquitin E3 ligases. Immunological Reviews. 2005; 207:112–25. 10.1111/j.0105-2896.2005.00314.x. 16181331

[42] Nathan JA , Lehner PJ . The trafficking and regulation of membrane receptors by the RING-CH ubiquitin E3 ligases. Experimental Cell Research. 2009; 315:1593–600. 10.1016/j.yexcr.2008.10.026. 19013150

[43] Manes TD , Hoer S , Muller WA , Lehner PJ , Pober JS . Kaposi’s Sarcoma-Associated Herpesvirus K3 and K5 Proteins Block Distinct Steps in Transendothelial Migration of Effector Memory CD4+ T Cells by Targeting Different Endothelial Proteins. J Immunol. 2010; 184:5186–92. 10.4049/jimmunol.0902938. 20357254 PMC2877909

[44] Schmidt K , Wies E , Neipel F . Kaposi’s sarcoma-associated herpesvirus viral interferon regulatory factor 3 inhibits gamma interferon and major histocompatibility complex class II expression. J Virol. 2011; 85:4530–7. 10.1128/JVI.02123-10. 21345951 PMC3126280

[45] Robey RC , Mletzko S , Gotch FM . The T-cell immune response against Kaposi’s sarcoma-associated herpesvirus. Adv Virol. 2010; 2010:1–9. 10.1155/2010/340356. 22331985 PMC3275983

[46] Roshan R , Labo N , Trivett M , Miley W , Marshall V , Coren L , Cornejo Castro EM , Perez H , Holdridge B , Davis E , Matus-Nicodemos R , Ayala VI , Sowder R , et al. T-cell responses to KSHV infection: a systematic approach. Oncotarget. 2017; 8:109402–16. 10.18632/oncotarget.22683. 29312617 PMC5752530

[47] Ma T , Patel H , Babapoor-Farrokhran S , Franklin R , Semenza GL , Sodhi A , Montaner S . KSHV induces aerobic glycolysis and angiogenesis through HIF-1-dependent upregulation of pyruvate kinase 2 in Kaposi’s sarcoma. Angiogenesis. 2015; 18:477–488. 10.1007/s10456-015-9475-4. 26092770 PMC4659376

[48] He QF , Xu Y , Li J , Huang ZM , Li XH , Wang X . CD81 T-cell exhaustion in cancer: Mechanisms and new area for cancer immunotherapy. Brief Funct Genomics. 2019; 18:99–106. 10.1093/bfgp/ely006. 29554204

[49] Gordon S , Plüddemann A . Tissue macrophages: Heterogeneity and functions. BMC Biology. 2017; 15:53. 10.1186/s12915-017-0392-4. 28662662 PMC5492929

[50] Halbrook CJ , Pontious C , Kovalenko I , Lapienyte L , Dreyer S , Lee HJ , Thurston G , Zhang Y , Lazarus J , Sajjakulnukit P , Hong HS , Kremer DM , Nelson BS , et al. Macrophage-Released Pyrimidines Inhibit Gemcitabine Therapy in Pancreatic Cancer. Cell Metab. 2019; 29:1390–1399.e6. 10.1016/j.cmet.2019.02.001. 30827862 PMC6602533

[51] Goossens P , Rodriguez-Vita J , Etzerodt A , Masse M , Rastoin O , Gouirand V , Ulas T , Papantonopoulou O , Van Eck M , Auphan-Anezin N , Bebien M , Verthuy C , Vu Manh TP , et al. Membrane Cholesterol Efflux Drives Tumor-Associated Macrophage Reprogramming and Tumor Progression. Cell Metab. 2019; 29:1376–1389.e4. 10.1016/j.cmet.2019.02.016. 30930171

[52] Wang J , Li D , Cang H , Guo B . Crosstalk between cancer and immune cells: Role of tumor-associated macrophages in the tumor microenvironment. Cancer Medicine. 2019; 8:4709–4721. 10.1002/cam4.2327. 31222971 PMC6712467

[53] United Republic of Tanzania, Ministry of Health and Social Welfare NACP. Guidelines on HIV Testing and Counseling in Clinical Settings. 2007 Available from http://www.who.int/hiv/topics/vct/TZ_PITC-Guidelines_ final edit_July2007.pdf.

[54] Tso FY , Kang G , Kwon EH , Julius P , Li Q , West JT , Wood C . Brain is a potential sanctuary for subtype C HIV-1 irrespective of ART treatment outcome. PLoS One. 2018; 13:e0201325. 10.1371/journal.pone.0201325. 30040863 PMC6057662

[55] Tso FY , Sawyer A , Kwon EH , Mudenda V , Langford D , Zhou Y , West J , Wood C . Kaposi’s Sarcoma-Associated Herpesvirus Infection of Neurons in HIV-Positive Patients. J Infect Dis. 2017; 215:1898–907. 10.1093/infdis/jiw545. 27932611 PMC5853869

