# Supplementary material for: Lack of CD8+ T-cell co-localization with Kaposi’s sarcoma-associated herpesvirus infected cells in Kaposi’s sarcoma tumors
Source: Oncotarget. 2020 Apr 28;11(17):1556–72. doi: 10.18632/oncotarget.27569 (PMC7197452; doi:10.18632/oncotarget.27569)
Supplement: Supplementary file 1 [file oncotarget-11-1556-s001.pdf]

Lack of CD8^{+} T-cell co-localization with Kaposi’s sarcoma-associated herpesvirus infected cells in Kaposi’s sarcoma tumors

SUPPLEMENTARY MATERIALS

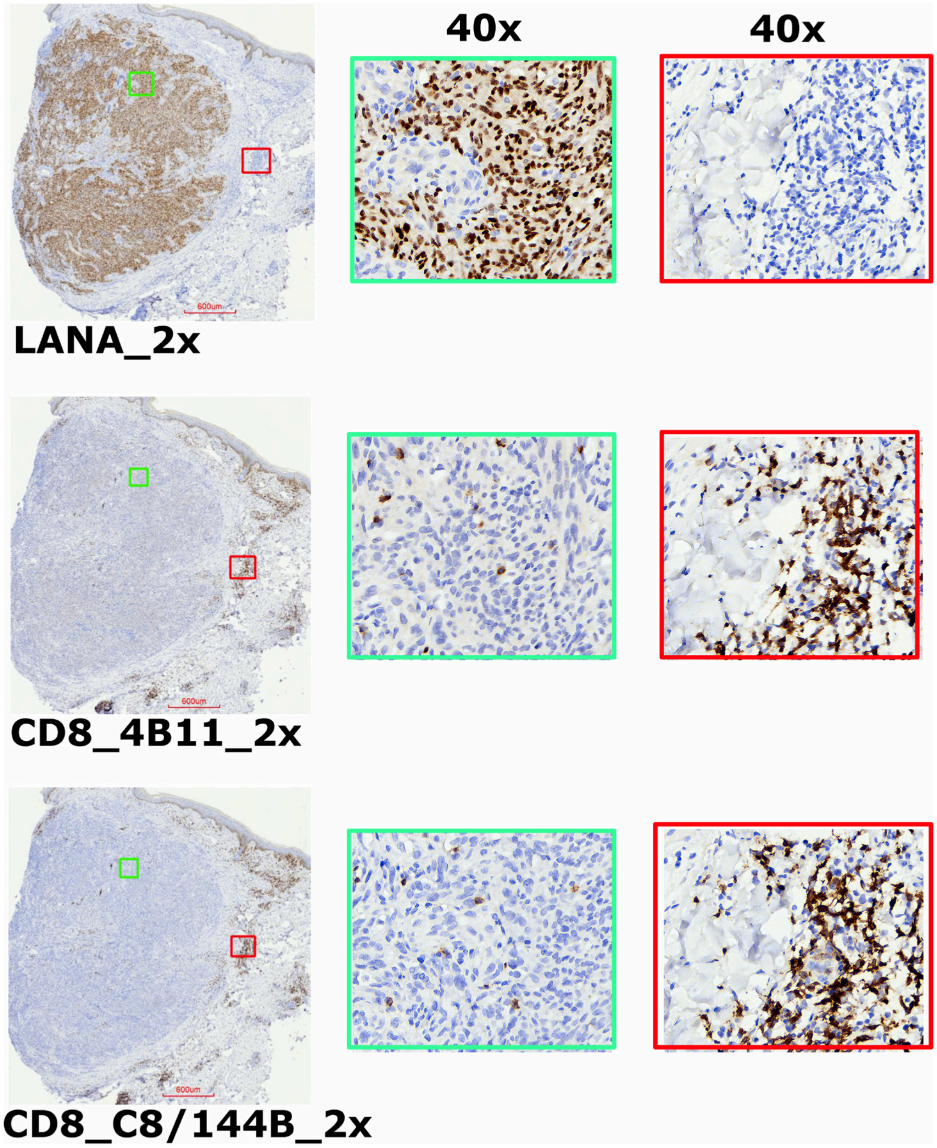

Supplementary Figure 1: Single-color immunohistochemical staining of adjacent KS tissue sections using mouse anti-LANA and two different clones of mouse anti-humanCD8 antibodies. Representative scanned images of adjacent sections stained for LANA, CD8 clone 4B11 and CD8 clone C8/144B at 2× magnification and 40× magnification of indicated regions ID-C3097. Green box – 40× magnified LANA+ region showing LANA+ or CD8+ T-cells. Red box – 40× magnified LANA- region showing LANA+ or CD8+ T-cells.

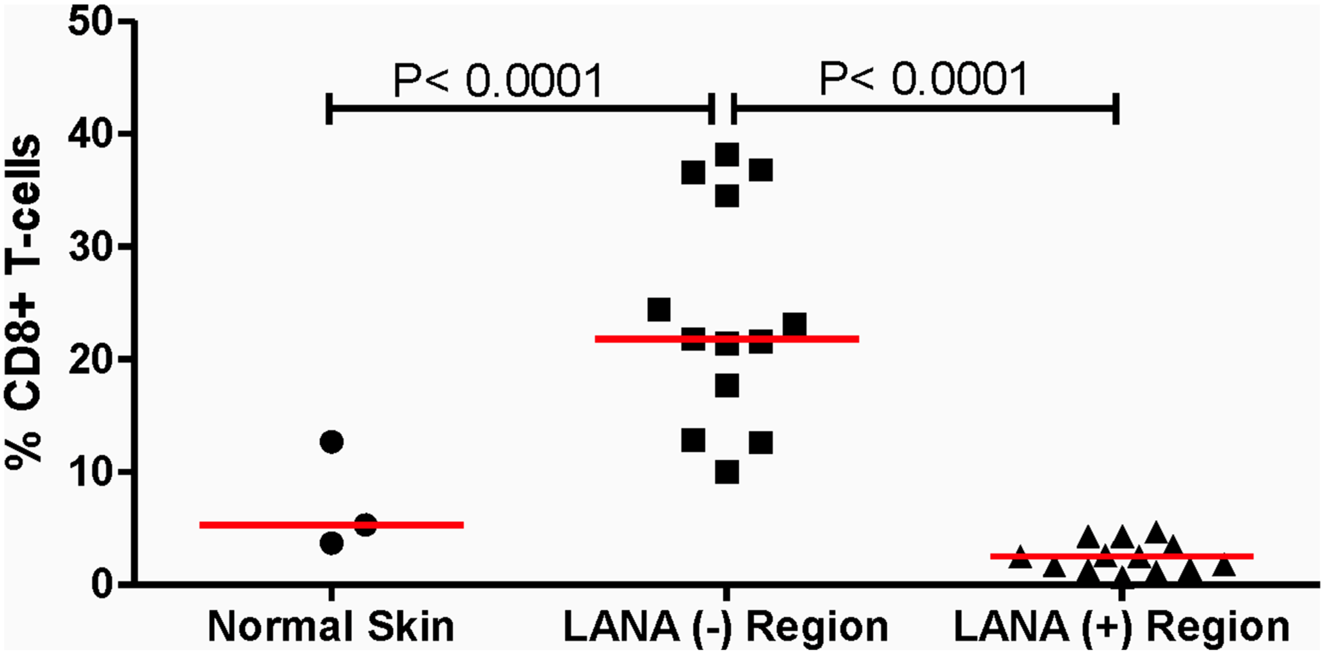

Supplementary Figure 2: Quantification plot for the percentage CD8 positive T-cells per field of view in dual-immunofluorescence images for normal skin, LANA- and LANA+ regions of KS biopsies. Red horizontal lines indicate median. (+) – Positive and (–) – Negative.
